# Expression Patterns of miR398, miR167, and miR159 in the Interaction between Bread Wheat (*Triticum* *aestivum* L.) and Pathogenic *Fusarium* *culmorum* and Beneficial *Trichoderma* Fungi

**DOI:** 10.3390/pathogens10111461

**Published:** 2021-11-11

**Authors:** Sylwia Salamon, Julia Żok, Karolina Gromadzka, Lidia Błaszczyk

**Affiliations:** 1 Department of Plant Microbiomics, Institute of Plant Genetics, Polish Academy of Sciences, 60-479 Poznan, Poland; ssal@igr.poznan.pl (S.S.); julka.zok666@gmail.com (J.Ż.); 2 Department of Chemistry, Poznan University of Life Sciences, 60-625 Poznan, Poland; karolina.gromadzka@up.poznan.pl

**Keywords:** wheat, plant–fungi interactions, microRNA, *Fusarium culmorum*, *Trichoderma atroviride*, *Trichoderma cremeum*, ddPCR

## Abstract

Bread wheat (*Triticum* *aestivum* L.) is an agronomically significant cereal cultivated worldwide. Wheat breeding is limited by numerous abiotic and biotic stresses. One of the most deleterious factors is biotic stress provoked by the *Fusarium* *culmorum* fungus. This pathogen is a causative agent of Fusarium root rot and Fusarium head blight. Beneficial fungi *Trichoderma atroviride* and *T. cremeum* are strong antagonists of mycotoxigenic *Fusarium* spp. These fungi promote plant growth and enhance their tolerance of negative environmental conditions. The aim of the study was to determine and compare the spatial (in above- and underground organs) and temporal (early: 6 and 22 hpi; and late: 5 and 7 dpi reactions) expression profiles of three mature miRNAs (miR398, miR167, and miR159) in wheat plants inoculated with two strains of *F. culmorum* (KF846 and EW49). Moreover, the spatial expression patterns in wheat response between plants inoculated with beneficial *T. atroviride* (AN35) and *T. cremeum* (AN392) were assessed. Understanding the sophisticated role of miRNAs in wheat–fungal interactions may initiate a discussion concerning the use of this knowledge to protect wheat plants from the harmful effects of fungal pathogens. With the use of droplet digital PCR (ddPCR), the absolute quantification of the selected miRNAs in the tested material was carried out. The differential accumulation of miR398, miR167, and miR159 in the studied groups was observed. The abundance of all analyzed miRNAs in the roots demonstrated an increase in the early and reduction in late wheat response to *F. culmorum* inoculation, suggesting the role of these particles in the initial wheat reaction to the studied fungal pathogen. The diverse expression patterns of the studied miRNAs between *Trichoderma*–inoculated or *F. culmorum*–inoculated plants and control wheat, as well as between *Trichoderma*–inoculated and *F. culmorum*–inoculated plants, were noticed, indicating the need for further analysis.

## 1. Introduction

Plants under attack from pathogens are able to manage and mediate the expression cascade of genes that activate and facilitate the host immune response. The endogenous small RNAs, including host microRNAs (miRNAs), are essential in the indicated reprogramming process. miRNAs are defined as non-coding single-stranded RNAs, 20–24 nt in length, and have a pivotal role in gene expression regulation at the posttranscriptional level [1,2]. The main mode of miRNAs’ action in plants relies on the cleavage of the open reading frame (ORF), complementary to the miRNA and degradation of the mRNA, resulting in translation failure. Alternatively, miRNA could bind to the 3′ untranslated region (3′UTR) and inhibit the initiation of translation [3]. miRNAs are involved in plants’ defense response against fungal [4], bacterial [5], nematode [6], and insect [7] invasions. During plant–pathogenic fungi interactions, miRNAs participate in two important defense mechanisms: PAMP-triggered immunity (PTI) triggered by pathogen-associated molecular patterns (PAMP) as a preliminary defense, and effector-triggered immunity (ETI) as a secondary defense, thereby regulating plant gene expression [8]. Moreover, miRNA molecules can be transmitted across kingdoms and can trigger gene silencing as trans-regulators in interacting, even evolutionary distant, organisms [9,10]. However, knowledge regarding miRNAs’ participation, regulation, and function in wheat–fungi interactions is still scarce.

Wheat is an agronomically important crop, with a worldwide production of 765 million tons [11]. Due to climate change and the need to provide global food security, wheat breeding should be the focus of interest not only from farmers but also from the scientific community. Understanding the sophisticated role of miRNAs in wheat–fungal interactions may initiate a discussion concerning the use of this knowledge to protect wheat plants from the harmful effects of fungal pathogens, including *Fusarium culmorum* (WG Smith) Sacc., which is the causative agent of Fusarium root rot (FRR) and Fusarium head blight (FHB). This species is also known as a postharvest pathogen, especially on freshly harvested grain that has not been dried or stored properly [12]. Yield and quality losses are particularly important when *F. culmorum* induces FHB, which develops from infection at anthesis and spreads until grain harvest, causing grain contamination with mycotoxins including estrogenic zearalenone and type B trichothecenes, such as deoxynivalenol, 3-acetyl-deoxynivalenol, nivalenol, and fusarenone X, which have a negative impact on human and animal health [12,13,14,15].

The highest antagonistic potential toward the mycotoxigenic *F. culmorum* has been proven in the *Trichoderma atroviride* AN35 strain [16]. This strain was also found to be an efficient producer of volatile metabolites, including antifungal 6-n-pentyl-2H-pyran-2-one [17] but a weak decomposer of plant cell wall degrading enzymes [18]. In contrast, *Trichoderma cremeum* AN392 strain has been characterized as an efficient producer of cellulases and xylanases [18] but with low mycoparasitic activity against *F. culmorum* [16]. *Trichoderma cremeum* was first described to be capable of secreting a 10-member lactone, named cremenolide (but-2-enoic acid 7-acetoxy-6-hydroxy-2-methyl-10-oxo-5,6,7,8,9,10-hexahydro-2H-oxecin-5-yl ester) characterized by the promotion of plant growth [19]. Meanwhile, recently, in both of these strains, *T. atroviride* AN35 and *T. cremeum* AN392, the possibility of establishing endophytic relationships with wheat plants—the colonization of surface and internal root tissues and their development—was found [20].

The knowledge of miRNAs’ involvement in wheat stress response is limited mainly to abiotic stresses. The miR398 has been shown to participate in *T. aestivum*’s response to cold and injury by affecting the target CSD gene responsible for the cell protection against the detrimental effects of superoxide radicals [21]. Interestingly, the abundance of miR398 is opposite in different types of biotic interaction. In *Citrus sinensis* plants inoculated with deleterious bacteria *Candidatus* Liberibacter asiaticus the down-regulation of miR398 expression was observed [22], whereas in the interaction Solanum lycopersicum–*Glomus intraradices* arbuscular mycorrhizal fungi, miR398 expression was upregulated [23]. miR167 has been documented to engage in wheat hybrid necrosis [24], as well as the response to salinity stress in *Tamarix chinensis* [25], drought in *Manihot esculenta* Crantz [26], and *Verticillium dahliae* infection in *Arabidopsis thaliana* [27]. Differences in the expression of miR159 were observed in the response of wheat plants to the presence of cadmium [28], salinity, cold [4], UV radiation [29], drought [30], and injury [21]. The target sequence of miR159 in wheat has been shown to be the gene encoding the MYB3 transcription factor, involved in signaling and development [28]. In addition, miR167 and miR159 were identified as responsive to powdery mildew infection caused by *Erysiphe graminis f.* sp. *tritici* (*Egt*), so these miRNAs may also participate in wheat biotic stress response [31]. However, nothing is known about possible communication by miR398, miR167, and miR159 between wheat and the pathogenic *F. culmorum* and the beneficial *Trichoderma* fungi.

Therefore, the aim of this study was to determine and compare the spatial (in above- and underground organs) and temporal (early: 6 and 22 hpi; and late: 5 and 7 dpi; reactions) expression profiles of selected miRNAs identified in Polish winter wheat Legenda plants inoculated with two strains (KF846 and EW49) of *F. culmorum* (miR398, miR167, and miR159). Moreover, the spatial expression patterns in wheat response between plants inoculated with *T. atroviride* AN35 strain and *T. cremeum* AN392 strain were assessed. In order to investigate differences between detrimental and beneficial wheat interactions on the miRNA level, the expression patterns of these molecules in the roots and leaves of plants treated with *Trichoderma* and *Fusarium* fungi in the late phase of the reaction were compared.

## 2. Results

The three studied candidate miRNAs, miR398, miR167, and miR159, were identified in the leaves and roots of wheat inoculated with pathogenic *F. culmorum* strains (EW49 and KF846) and plant beneficial species: mycoparasitic *T. atroviride* AN35 and saprophytic *T. cremeum* AN392.

### 2.1. Interactions between Triticum aestivum L. and Selected Fungi

**Figure 1:**
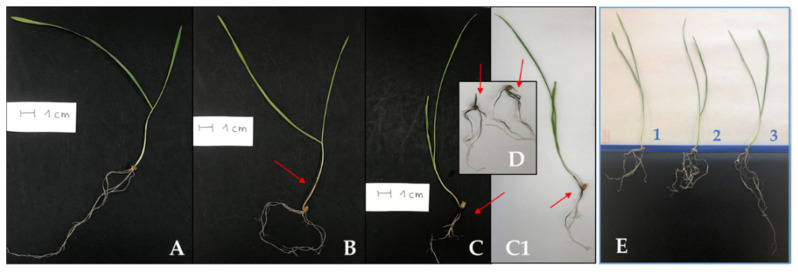
Examples of 14-day-old wheat seedlings: (**A**) 7 days after treatment with water, healthy control plant; (**B**) 7 days after infection with *F. culmorum* EW49 with slightly pink-beige discoloration of the stem; (**C**,**C1**,**D**) 7 days after infection with *F. culmorum* KF846 with necrotic symptoms manifested by a brownish-purple color of the roots and stems. An arrow marks necrotic changes on the roots and stems of seedlings; (**E**, seedling No 1) 7 days after treatment with water, control plants; (**E**, 2 seedling No 2) 7 days after treatment with *T. atroviride* AN35; (**E**, seedling No 3) 7 days after treatment with *T. cremeum* AN392.

**Figure 2:**
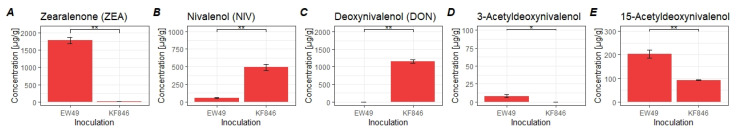
Mycotoxin analysis of zearalenone (**A**), nivalenol (**B**), deoxynivalenol (**C**), 3-acetyldeoxynivalenol (**D**), and 15-acetyldeoxynivalenol (**E**) in *F. culmorum* EW49 and KF846 strains. Means ± SD; significant differences were marked as follows: **—*p* ≤ 0.01; *—*p* ≤ 0.05.

The detrimental interactions were studied at the early (6 and 22 hpi) and late (5 and 7 dpi) stages after *F. culmorum* inoculation. Seven days after *F. culmorum* inoculation, the wheat plants showed the symptoms of foot and root rot (FRR) disease, with tissue lesions: on a scale of 1 browning index BI (slightly necrotic, beige discoloration of the roots and stems) observed in 40% of seedlings and on a scale of 0 (without symptoms) observed in 60% of seedlings after inoculation with EW49; on a scale of 4 (completely necrotic, a brownish-purple color of the roots) observed in 5% of seedlings, on a scale of 2–3 (from moderately necrotic to severely necrotic) observed in 35% of seedlings, on a scale of 1 (slightly necrotic, and slightly beige discoloration of the roots) observed in 40% of seedlings and on a scale of 0 observed in 20% of seedlings after inoculation with KF846 (Figure 1A–D). Mycotoxin analysis showed the ability of both strains to produce ZEA, NIV, DON, and 15-Ac-DON on a solid rice medium, with the EW49 strain additionally producing 3-Ac-DON. The quantitative analysis showed differences in the concentrations of these toxins; in the EW49 cultures, a higher concentration of ZEA and 15-Ac-DON was observed, while in the KF846 cultures, a higher concentration of NIV and DON was noted (Figure 2).

The beneficial relationship between wheat seedlings and *Trichoderma* spp. was studied on day 7 after inoculation. In this time interval, changes in the root architecture of plants treated with both *T. atroviride* and *T. cremeum* were observed, whereby these changes were different for the studied species. These effects are presented in Figure 1E.

To check the specificity of the used assays, two types of negative control were applied in ddPCR analysis: regular no template control (NTC_1), as well as a control consisting of stem-loop pulsed RT-PCR product without template (NTC_2). The NTC_1 control was negative in all the studied assays; however, the NTC_2 showed a few positive signals in ddPCR in the case of miR398 but at an approximated level in all the studied groups, and thus, we decided to use the designed assay for miR398 analysis.

### 2.2. Early Response of Wheat’s miR398, miR167, and miR159 to Fusarium culmorum Infection

**Figure 3:**
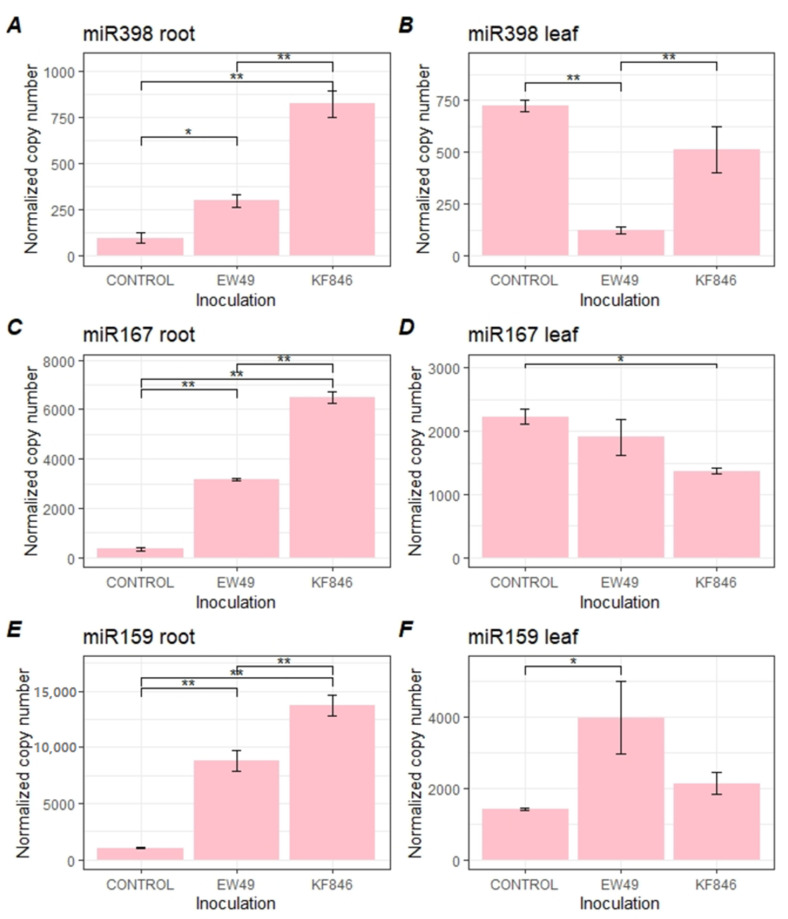
Expression levels of miR398 (**A**,**B**), miR167 (**C**,**D**), and miR159 (**E**,**F**) in the roots and leaves of bread wheat during the early stage (6 and 22 hpi) after inoculation with two strains of *F. culmorum* (EW49, KF846) and in control plants (wheat without fungal inoculation). Means ± SD; significant differences were marked as follows: **—*p* ≤ 0.01; *—*p* ≤ 0.05.

The abundance of all analyzed miRNAs in the studied roots increased at the early wheat response after *F. culmorum* inoculation (Figure 3, Table S1). Moreover, the differences between EW49 and KF846 inoculation were observed in miR398, miR167, and miR159 abundance. In all studied miRNAs in roots, the KF846 strain induced a higher expression level in comparison to EW49. In leaves, the expression profiles of miR398, miR167, and miR159 were diverse (Figure 3, Table S1).

*Fusarium culmorum* EW49 caused a significant decrease in the miR398 level compared to the control (*p* ≤ 0.01), as also seen in KF846 plants (*p* ≤ 0.05), whereas miR159 was more abundant after EW49 inoculation (*p* ≤ 0.05). The miR167 level was decreased in leaves treated with KF846 in relation to control plants. miR398 was the least abundant studied miRNA in the roots and leaves of bread wheat. We also observed a distinct level of studied miRNAs between the roots and leaves of control plants. In general, miR398 and miR167 were more abundant in the leaves of healthy plants.

### 2.3. Late Response of Wheat’s miR398, miR167, and miR159 to Fusarium culmorum Infection

**Figure 4:**
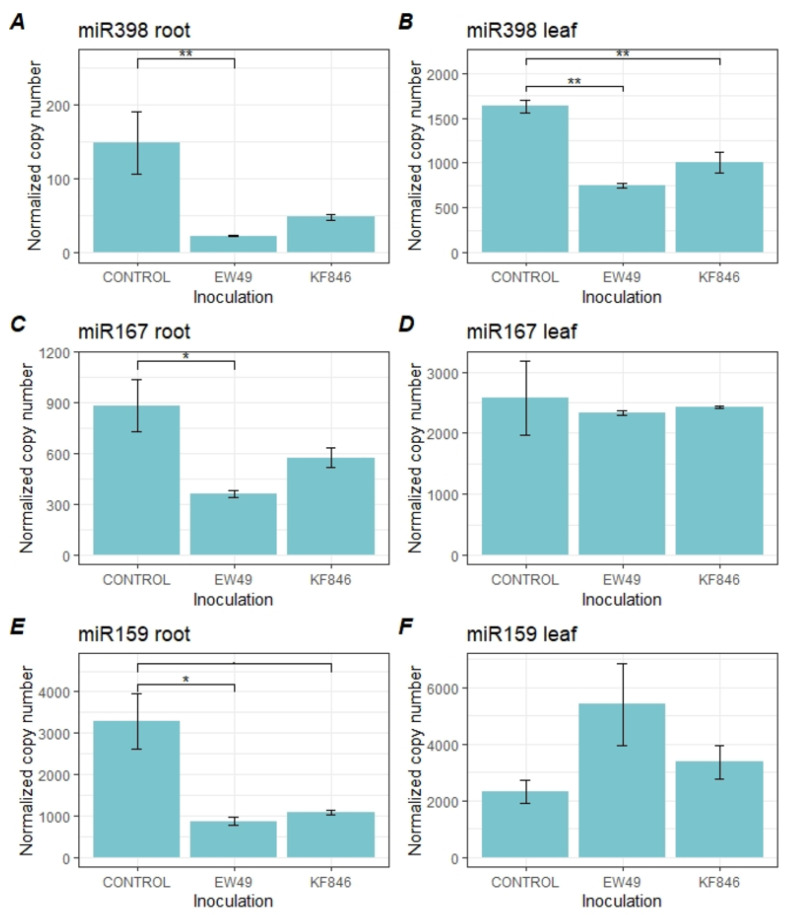
Expression levels of miR398 (**A**,**B**), miR167 (**C**,**D**), and miR159 (**E**,**F**) in roots and leaves of bread wheat during the late stage (5 and 7 dpi) after inoculation with two strains of *F. culmorum* (EW49, KF846) and in control plants (wheat without fungal inoculation). Means ± SD; significant differences were marked as follows: **—*p* ≤ 0.01; *—*p* ≤0.05; unmarked line—*p* ≤ 0.1.

At the late stage after EW49 inoculation (5 and 7 days) a significant reduction in miR398, miR167, and miR159 was noticed in roots (Figure 4, Table S2). During this period, the level of miR398 in wheat leaves was lower both after inoculation with EW49 and after KF846. It was observed that *F. culmorum* infection did not have any impact on miR167 and miR159 levels in leaves.

### 2.4. Comparison of the Temporal Response of Wheat’s miR398, miR167, and miR159 to Fusarium culmorum Infection

**Figure 5:**
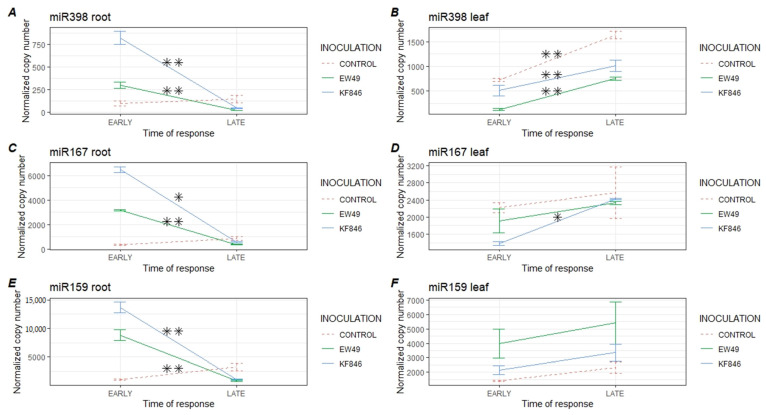
Comparison of the expression levels of miR398 (**A**,**B**), miR167 (**C**,**D**), and miR159 (**E**,**F**) in roots and leaves of bread wheat during the early (6 and 22 hpi) and late (5 and 7 dpi) response on inoculation with two strains of *F. culmorum* (EW49, KF846) and in control plants (wheat without fungal inoculation). Means ± SD; significant differences were marked as follows: **—*p* ≤ 0.01; *—*p* ≤ 0.05.

A significant reduction in the expression levels of studied miRNAs was observed in the late response of wheat roots to both *F. culmorum* EW49 and KF846 inoculation (Figure 5, Table S3), However, the abundance of miR398 increased with time in the leaves of the plants inoculated with EW49 and KF846 and in the group of control plants. This may indicate miR398’s participation in development or other processes that occurred in wheat leaves. Similarly, the expression of miR167 in the leaves of plants infected with KF846 increased over the time analyzed, reaching the level noted in the control plants. The amount of miR159 in wheat leaves was similar in the early and late stage of wheat reaction in all studied groups.

### 2.5. Analysis of the Response of Wheat miR398, miR167 and miR159 to Inoculation of Trichoderma Species and Comparison to the Profiles Formed by Pathogenic Fusarium Strains

**Figure 6:**
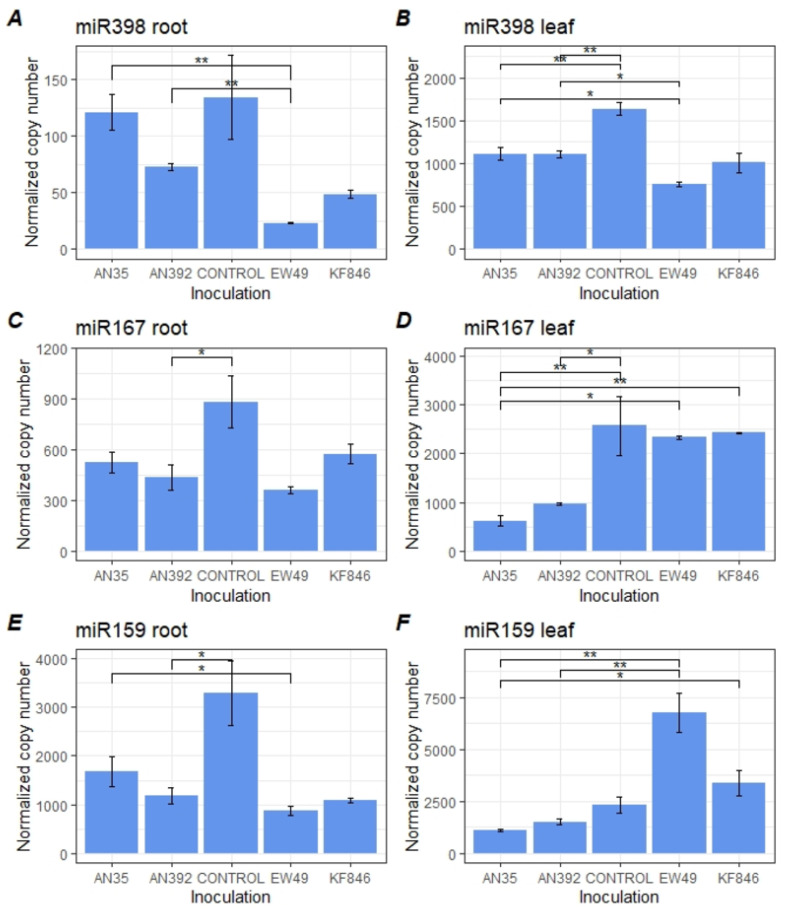
Comparison of the expression levels of miR398 (**A**,**B**), miR167 (**C**,**D**), and miR159 (**E**,**F**) in roots and leaves of bread wheat in response to *T. atroviride* (AN35), and *T. cremeum* (AN392) treatment and to late phase infection with pathogenic *F. culmorum* strains (EW49, KF846) and to control plants (wheat without fungal inoculation). Means ± SD; Only the significant differences observed between *Trichoderma*–inoculated plants vs. *Fusarium*–inoculated plants and control wheat plants were marked as follows: **—*p* ≤ 0.01; *—*p* ≤ 0.05.

A significant reduction in miR167 and miR159 was observed in *T. cremeum* AN392 colonized wheat roots (Figure 6). A reduction in the expression level of miR167 was also observed in wheat leaves after inoculation with *T. cremeum* but also after treatment with *T. atroviride*. Both of these species also inhibited miR398 expression in wheat leaves. The differences in the expression of the studied miRNAs between wheat plants inoculated with *Trichoderma* species and those treated with *Fusarium* strains in the late phase of cointeraction were also analyzed (Figure 6). The level of miR398 in roots and leaves diverged between the plants inoculated with *Trichoderma* (AN35, AN392) and those inoculated with *F. culmorum* EW49. Accumulation of miR167 in roots was similar between wheat plants inoculated with *Trichoderma* and *Fusarium* but significantly lower in wheat leaves after *Trichoderma* inoculation than after pathogen infection. The amount of miR159 was significantly higher in roots of plants treated with AN35 strain than in roots of plants inoculated with *F. culmorum* EW49. Moreover, a significant reduction in miR159 expression was observed in leaves of AN35-treated plants in relation to *Fusarium*–inoculated plants as well as in leaves of AN392-treated plants in relation to KF846-inoculated plants (Figure 6, Table S4).

## 3. Discussion

miR398, miR167, and miR159 are conserved miRNAs that participate in the plant’s response to abiotic and biotic stresses. Previous research showed that miR398 cleaves *CSD* mRNA in wheat [32] and downregulates the *CSD1* and *CSD2* genes coding cytosolic and chloroplastic Cu-Zn-type superoxide dismutases (Cu/Zn-SOD), respectively [33,34,35]. It is worth mentioning that this group of metalloenzymes are known to play a vital role in the detoxification of reactive oxygen species (ROS) and act as dismutase by catalyzing superoxide (O2^−^) to molecular oxygen (O2) and H2O2 [36]. As various environmental changes and stress conditions can stimulate ROS production, superoxide dismutases (SODs) are therefore considered to be part of the mechanism involved in plant defense strategy. It has been documented that SOD proteins in wheat are employed both in biotic and abiotic stress responses. Recently, Tyagi et al. [37] supported the participation of *TaCu-ZnSOD* genes in the response of wheat to biotic and abiotic stress by analyzing their expression after the infection of plants with *Blumeria graminis* and *Puccinia striiformis* and in conditions of heat, drought, and salinity. With regard to Li et al.’s [33] research proving the participation of miR398 in the regulation of the CSD genes expression, the role of this molecule in modulating wheat response to oxidative stress, and thus in the adaptation to unfavorable environmental conditions, has been recognized [23,30,32,38,39,40,41,42]. However, miR398 in wheat has been identified mostly in response to abiotic stresses such as cold [23,32], injury [23], and drought [32,42]. The results of our research suggest that miR398 is also involved in wheat responses to infections with pathogenic *F. culmorum* strains, as well as inoculation with symbiotic *Trichoderma* fungi.

This study revealed that the level of miR398 in wheat roots increased significantly in the early stages of *F. culmorum* infection and decreased several days after inoculation. In the leaves, however, the situation was completely opposite; in the early phase of the interaction of wheat–*Fusarium*, the expression of miR398 was significantly lower than in the later phase. This may indicate a systemic reaction of the plant to infection with the pathogenic *F. culmorum*. There were also slight differences between the *Fusarium* strains in their effect on miR398 levels, both in the roots and in the leaves, and in the early and late phase of interaction with wheat. Therefore, miR398 biosynthesis is believed to depend not only on the interaction time of these fungi with the wheat seedlings and its organs but also on the *F. culmorum* strain. Similar conclusions were reached by Tyagi et al. [37], whose research concerned the analysis of the expression of *TaCu-ZnSOD* genes in the early (24 hpi) stages of wheat infection by *B. graminis* and *P. striiformis*. The examined genes showed modulated expression, and their patterns appeared to be pathogen-specific [37]. However, to confirm these assumptions in our experimental system, wheat–*F. culmorum*, further research is needed, including analysis of the expression of a potential target gene for miR398. As already mentioned, analyzing the individual levels of miR398 in the experimental systems studied here, it was observed that the expression of miR398 in the roots of plants treated with *Fusarium* decreased over time, while in the leaves of pathogen-treated plants, the expression of this molecule increased; however, its level both in the early and late phase of *Fusarium* interaction with wheat was lower than in the leaves of the control plants. Observations of reduced miR398 expression in leaves were observed in *Oryza sativa* seedlings inoculated with *Xanthomonas oryzae* pv. *oryza* and *Magnaporthe grisea* [38], as well as in leaves of *A. thaliana* inoculated with *Pseudomonas syringae* [39] and in leaves of *Solanum lycopersicum* infected with *Phytophthora infestans* [40]. To the best of our knowledge, this is the first report to analyze the expression profile of miR398 in bread wheat’s response to *F. culmorum*.

So far, nothing has been known about the involvement of miR398 in interactions between wheat and the symbiotic *Trichoderma*. We assessed the abundance of miR398 in the late phase (7 dpi) of wheat’s interaction with this beneficial fungus and demonstrated a reduction in its expression in leaves, after treatment with both *T. atroviride* and *T. cremeum*. However, we did not notice significant differences in the level of this molecule in the roots of plants treated with these fungi. On the other hand, Naya et al. [41] observed downregulation of miR398 expression in roots at the early stage (24 hpi) of interaction between common bean and the symbiotic bacteria *Rhizobium tropici*, but the authors did not analyze the later stage of these interaction, so we cannot compare these results with our findings. Therefore, in order to observe the role of this particle in the symbiotic interactions of wheat with fungi, there is a need for more detailed analyses of miR398, also in the early phase of the interaction, and especially its target evaluation in the *Trichoderma* inoculated wheat plant.

miR167 as well as miR159 regulate the hormone signaling pathway of their targets, which are transcription factors. miR167 targets auxin response factor 8 (ARF8) in wheat and indole-3-acetic acid-alanine resistant3 (IAA-IAR3) in *A. thaliana* [43,44,45,46]. ARF genes are involved in the regulation of auxin response genes [45]. It has been demonstrated that some plant pathogens produce auxin-like compounds or change the auxin signaling in plants and consequently increase the host’s susceptibility to infection [47,48]. For example, in wheat, 15 ARF genes were differentially expressed in leaves during *Puccinia triticina* invasion [49], suggesting that these genes may be under the post-transcriptional control of miRNAs. miR159 targets MYB33 transcription factor in wheat [43]. It has been proven by Reyes and Chua [50] that the accumulation of miR159 is induced by abscisic acid (ABA) and leads to the degradation of MYB33 and MYB101 transcripts in *A. thaliana* seedlings, indicating the participation of miR159 in the ABA signaling transduction pathway and its negative regulation in the ABA responses. Meanwhile, Gupta et al. [4] reported that miR159 levels in wheat decreased during cold and salt stress and increased during drought. However, as a result of infection with *Fusarium oxysporum*, Pavitra et al. [51] observed less accumulation of miR159 in banana roots. The knowledge about miRNAs’ participation in *F. culmorum*–wheat interaction is very limited. Inal et al. [52] using miRNA microarrays observed a decrease in miR159 levels in resistant wheat cultivars and its increase in susceptible plants after a 14-day infection with *F. culmorum*. In the work presented here, we observed that miR167 and miR159 levels increased in wheat roots at the early stage of *F. culmorum* infection and decreased a few days after inoculation. These results are consistent with the response of wheat to another biotic stress, namely infection by *Puccinia graminis* f. sp. *tritici* [53]. In the studies of Gupta et al. [53], two susceptible wheat cultivars showed an increase in miR167 and miR159 levels in the early stage of response (2dpi) and a decrease in the late stage (10 dpi). Cultivars moderately resistant to *P. graminis* infection demonstrated an increased level of miR167 and a very low amount of miR159. Meanwhile, susceptible cultivars presenting more acute symptoms demonstrated a higher level of these miRNAs than resistant wheat. In our research, higher accumulation of miR167 and miR159 was also observed in plants with symptoms of FRR, which occurred as a result of infection with a very harmful strain of *F. culmorum* KF846. Gupta et al. [53] suggested that increase in the levels of the specific miRNAs in the early stages of infection and their decrease in the later stages of disease symptoms development may be a part of the mechanism of neutralizing the hormonal imbalance caused by fungal invasion in wheat cells, or it may be the initiation of the host defense response through the signaling pathway. Recently, Jin et al. [54] using the small RNA-seq approach identified the miRNAs involved in *Fusarium graminearum* infection in wheat at 30 and 50 hpi. Interestingly, miR398, miR167, and miR159 did not belong to the 26 miRNAs showing differentiated expression in spikes, but were identified in the studied material. The findings suggest that miRNA involvement is crucial, especially at a very early stage (<30 hpi) after contact with the pathogen in the colonizing plant tissue.

Interestingly, the levels of miR167 and miR159 in the roots of *T. cremeum*–inoculated plants were significantly lower than those observed in the control plants. On the other hand, in leaves, the miR167 level was significantly lower in plants treated with *T. cremeum* or *T. atroviride*, and the miR159 level was similar to that recorded in the control plants. It is also worth noting that the biosynthesis of both these particles in wheat leaves after *Trichoderma* inoculation was lower than after *Fusarium* inoculation. It has been previously documented that lower levels of miR167 lead to overexpression of auxin response genes [55]. It can therefore be assumed that the lower level of miR167 observed here is related to the plant growth-stimulating properties of *Trichoderma* fungi. This is another aspect worth unraveling by carrying out additional analyses, mainly examining the expression of miR167 target genes, in order to confirm this suggestion. Meanwhile, our current results show that miR167 and miR159 are involved in symbiotic wheat–*Trichoderma* interactions, and that their expression profiles appear to be spatially and species-specific. So far, only Ye et al. [25] investigated the role of these miRNAs in interactions between plants and beneficial microorganisms. These authors noted, after 8 weeks of the interaction of *Oncidium orchids* with *Piriformospora indica*, increased regulation and no differences in the levels of miR167 and miR159 in the roots, respectively [25].

The comparison between wheat response to two different pathogenic *F. culmorum* strains, KF846 and EW49, was conducted. The strains differed in ability to produce mycotoxins (higher concentration of ZEA and 15-Ac-DON in the EW49 strain and higher concentration of NIV and DON in the KF846 strain), as well as in the distinct level of tissue lesions caused (EW49: 0–1; KF846: 2–4). We observed that inoculation with the KF846 strain, which is more detrimental for wheat, resulted in greater accumulation of miR398, miR167, and miR159 in wheat roots at the early stage of the reaction than in the case of EW49; however, in both cases, the level of these molecules was higher compared to control plants. These differences indicate strain-specific miRNAs abundance in bread wheat. We also observed diverse expression profiles between roots and leaves of each miRNA in *Fusarium*–inoculated plants, but in the late stage, the evaluated patterns were similar. In addition, there were also differences in miRNAs’ abundance between roots and leaves. The results of the recent literature do not indicate that the roles of studied miRNAs in roots and leaves of wheat are different; however, our observations in miR398 leaves (increased miR398 abundance in control plants) suggest that the alternative target transcript is controlled by these molecules, and points out the need for further investigation of the role of miR398 in leaves. Comparison of these results with expression level of transcripts controlled by miR398, miR167, and miR159 is needed to understand their role in *F. culmorum* pathogenesis.

## 4. Materials and Methods

### 4.1. Plant Material and Growth Conditions

The material used for the experiment was the roots and leaves of bread winter wheat Legenda developed by Poznań Plant Breeders Ltd. (Poland). Wheat seeds were surface sterilized with 70% ethanol, followed by 5% sodium hypochlorite, washed with sterilized distilled water, and germinated on moist sterile filter papers to screen for microbial contamination. After seven days’ incubation at 23 °C, the seedlings with a similar root and leaf length were selected to be placed in the experimental (5 × 5 × 5 cm) pots filled with autoclaved quarts sand and grown in a phytotron under controlled (optimal) conditions with a photoperiod of 10/14 h (day/night), 60% ± 5% relative humidity, and 22/20 ± 1 °C temperature. The experiment was arranged in a completely randomized design with five treatments (distilled water—control, *F. culmorum* KF846, *F. culmorum* EW49, *T. atroviride* AN35, *T. cremeum* AN392 inoculation), four (*Fusarium*)/one (*Trichoderma*) time points, three pot-replications, and 8 plants per sample.

### 4.2. Fungal Collection

*Fusarium culmorum* KF846, *F. culmorum* EW49, *T. atroviride* AN35, and *T. cremeum* AN392 strains are a part of the collection of the Institute of Plant Genetics of the Polish Academy of Sciences. Strain KF846 was isolated from bread wheat kernels, and strain EW49 was isolated from the rhizosphere of spelt wheat (*T. aestivum* ssp. *spelta*). Both strains of *F. culmorum* were characterized for their production of mycotoxins and their ability to cause foot and root rot (FRR) disease in wheat.

*Trichoderma atroviride* AN35 was isolated from maize grains and characterized as the *Fusarium* mycoparasite with the highest ability to form volatile metabolites [17,20,56,57]. *Trichoderma cremeum* AN392 was isolated from pieces of decaying wood and characterized as an efficient producer of cellulases and xylanases [18]. Both studied *Trichoderma* strains have recently been recognized as effective colonizers of wheat roots and their opportunistic symbionts [20].

### 4.3. Mycotoxin Analysis

The toxicogenic capacity of *F. culmorum* strains was determined on the basis of solid medium cultivation on rice as described by Błaszczyk et al. [16]. Zearalenone (ZEA), nivalenol (NIV), deoxynivalenol DON, 3-acetylo-deoxynivalenol (3-Ac-DON) and 15-acetylo-deoxynivalenol (15-Ac-DON) were extracted from a 21-day dried fungal culture on rice with an acetonitrile:water (90:10, *v*/*v*) solution using a solvent mixture in a ratio of 2.5 mL of solvent per 1 g of ground samples, and then they were homogenized [16]. The mycotoxins were determined by using a chromatographic system according to the appropriate methods: (a) Zearalenone content was determined using a Waters 2695 high performance liquid chromatograph, a Waters 2475 Multi λ Fluorescence Detector, and a Waters 2996 Array Detector (Waters Corporation, Milford, MA, USA). The excitation and emission wavelengths were 274 and 440 nm, respectively. The reverse-phase column was a C-18 Nova Pak column (3.9 × 150 mm), while the mobile phase was acetonitrile:water:methanol (46:46:8, *v*/*v*/*v*), at a flow rate of 0.5 mL min^−1^. Quantification of ZEA was performed by measuring the peak areas at the ZEA retention time according to the relevant calibration curve (correlation coefficient R = 0.9998). The limit of zearalenone detection was 3 μg kg^−1^. The recovery rate of ZEA was from 97% to 99%, estimated in triplicate by extracting mycotoxins from blank samples spiked with 5–100 ng g^−1^ of the compound. The relative standard deviation (R.S.D.) was below 1% for ZEA; (b) Trichothecenes were quantified using a Waters 2695 apparatus with a C-18 Nova Pak column (3.9 × 300 mm) and a Waters 2996 Array Detector (λmax = 224 nm for DON and NIV). Mycotoxins were eluted from the column with a 25% water solution of methanol (flow rate 0.7 mL min^−1^) with retention times of 11.72 and 7.46 min, respectively. The detection limit was 0.01 μg g^−1^. The results (on the basis of retention times) were confirmed by comparison with the relevant calibration curve (correlation coefficients R = 0.9997). Recovery rates of NIV, DON, 3-AcDON, and 15-Ac-DON were 75%, 87%, 76%, and 75, respectively, estimated in triplicate by extracting mycotoxins from blank samples spiked with 10–100 ng g^−1^ of the compounds. The relative standard deviation (R.S.D.) was below 5%. Mycotoxin standards and reagents for analysis were supplied by Sigma-Aldrich (Steinheim, Germany).

### 4.4. Inoculum Preparation and Plant Inoculation

Pure cultures of fungal strains were recultured from stock slants onto 8.5 cm-diameter Petri dishes and were grown on potato dextrose agar (PDA) medium for 7 days at 25 ± 2 °C and, subsequently, were used as the inoculum. Using stainless steel cork bore (Bochem™ Stainless Steel Cork Bore, Bochem Instrumente GmbH, Weilburg, Germany), a 4 mm-diameter agar disc with sporulating mycelium, cut from the center of the 7-day culture, was placed at the base of the shoot of each 10-day-old wheat seedling growing in quartz sand. One 4 mm agar disc covered with sporulating mycelium contained on average 2 × 10^5^ *Fusarium* spores mL^−1^ or 5 × 10^5^ *Trichoderma* spores, determined with a hemocytometer by scraping the spore layer from the agar disc surface.

### 4.5. Sampling, Disease Symptoms Assessment

For analysis, leaves and roots of eight wheat seedlings were collected for each time point and for each treatment. The seedlings inoculated with *F. culmorum* strains were harvested at 6 and 22 hpi (hours post inoculation), as well as 5 and 7 dpi (days post inoculation). At 7 dpi, after sampling the roots and the aerial parts of the plants inoculated with the *F. culmorum* strains, the visual symptoms of the disease were assessed according to the index described by Beccari et al. [58], namely the browning index (BI), on a scale from 0 (no symptoms) to 4 (completely necrotic). The seedlings inoculated with beneficial *Trichoderma* fungi were collected at 7 dpi. The choice of this time point was determined by changes in the root architecture (Figure 1) and changes in the level of genome function (unpublished transcriptomic, proteomic, and metabolomic data) observed in plants 7 days after treatment with *Trichoderma* species.

### 4.6. Total RNA Isolation

RNA isolation from 100 mg of plant tissues was performed using the Direct-zol RNA MiniPrep kit dedicated to purifying high-quality total RNA, including 17–200 nt-long small RNAs (Zymo Research, Irvine, CA, USA) and Plant Isolation Aid (Thermo Fisher Scientific, Waltham, MA, USA ), according to the protocol described by Smoczynska et al. [59]. The RNA quality was analyzed by electrophoresis in 1.5% agarose gel, and the concentration of the obtained RNA was measured spectrophotometrically using Nanodrop (ThermoFisher Scientific, Waltham, Massachusetts, USA). For plant samples inoculated with *F. culmorum* strains, equivalent amounts of isolated RNA representing different collection times were pooled and two groups of samples corresponding to the early (6 and 22 hpi) and late (5 and 7 dpi) responses were formed. Pooled RNA samples were prepared in three biological replicates.

### 4.7. Stem Loop Pulsed RT-PCR

To detect candidate miRNAs, the stem loop pulsed RT-PCR method was applied [60], followed by absolute quantification using droplet digital PCR (Bio-Rad Laboratories, Inc, Hercules, CA, USA). The primers for miR398 were designed using the Stem Loop Primers Designer tool (http://www.flujogenico.cl/plantbiotech/amir319edesigner/stemloop/, accessed on 7 January 2020). For the analysis of miR167 and miR159, the primers described by Feng et al. [61] were applied. Nucleotide sequences of the studied miRNAs, as well as stem loop primer sequences, are presented in Table 1.

Stem loop pulsed RT-PCR was performed with 10 ng of total RNA. RNA was added to the reaction mixture prepared as follows: 1 µL of denatured 0.3 µM stem loop primer, 0.5 µL of 10 mM dNTP (Sigma Aldrich, Saint Louis, MO, USA), 0.1 µL of Super Script III RT 200U/µL (Thermo Fisher Scientific, MA, USA), 4 µL of 5× First Strand Buffer, 2 µL of 0.1 M DTT, 0.1 µL of RNAse OUT 40U/µL (Thermo Fisher Scientific, Waltham, MA, USA), and RNAse-free water up to 20 µL. The reaction was carried out in a T-100 thermocycler (Bio-Rad Laboratories, Inc, Hercules, CA, USA) in the following conditions: 30 min incubation at 16 °C followed by 60 cycles at 30 °C for 30 s, 42 °C for 30 s, and 50 °C for 1 s. The reaction was finished by 5 min incubation at 85 °C in order to inactivate the reverse transcriptase.

### 4.8. Reverse Transcription

To normalize the levels of selected miRNAs, the ADP-ribosyltransferase gene was used. For reference gene analysis, the 1300 ng of total RNA was reverse transcribed with the use of the iScript™ cDNA Synthesis Kit (Bio-Rad Laboratories, Inc., Hercules, CA, USA) according to the manufacturer’s instruction. The reaction was performed in T-100 thermocycler (Bio-Rad Laboratories, Inc, Hercules, CA, USA) under the following conditions: (1) 25 °C for 5 min, (2) 46 °C for 20 min, and (3) 95 °C for 1 min), followed by 40-fold dilution of the obtained cDNA.

### 4.9. Droplet Digital PCR

Absolute quantification of selected miRNAs, as well as the reference gene, was carried out using the EvaGreen Digital PCR Supermix (Bio-Rad Laboratories, Inc., Hercules, CA, USA) and Droplet Digital PCR System (Bio-Rad Laboratories, Inc, Hercules, CA, USA). Primer sequences applied in this analysis are listed in Table 2. ddPCR mixture was performed with the use of 2 µL of cDNA after Stem loop pulsed PCR, 1× Eva Green Digital PCR Supermix, 114 nM of each primer, RNAse-free water up to 22 µL, and QX200™ Droplet Generation Oil for EvaGreen (Bio-Rad Laboratories, Inc, Hercules, CA, USA) using a QX200 Droplet Generator (Bio-Rad Laboratories, Inc., Hercules, CA, USA). Amplification was carried out by the T100 Thermal Cycler (Bio-Rad Laboratories, Inc., Hercules, CA, USA) according to the following conditions: (1) 95 °C for 5 min, (2) 95 °C for 30 s, (3) 58 °C for 1 min, steps 2–3 × 40, (4) 4 °C for 5 min (5) 90 °C for 5 min. For all steps, a ramp rate of 2 °C/s was applied. Finally, the fluorescence of PCR products was measured using a QX200TM Droplet Reader (Bio-Rad Laboratories, Inc., Hercules, CA, USA). Plate designing was performed using QuantaSoftTM Analyses Pro Software program (Bio-Rad Laboratories, Inc., Hercules, CA, USA). For all examined miRNAs, two types of negative controls were applied: regular no template control (NTC_1), as well as a control consisting of a stem loop pulsed RT-PCR product without a template (NTC_2).

### 4.10. Statistical Analysis

The obtained results were normalized and presented as normalized copy numbers for every 100 copies of the reference gene. A few of the outlier measurements were removed from the analysis. The statistical analysis of the obtained results was carried out using R version 3.6.3. [62]. The anova or kruskal.test functions were used depending on the results of the Bartlett test for homogeneity of variance using the bartlett.test function (stats package) [62]. When the statistics indicated that inoculation affected miRNA expression, a Tukey post hoc test was performed using the TukeyHSD function from the stats package [62], or the Dunn test [63] from the FSA package v. 0.8.32 [64] to observe which groups presented significant differences.

**Table 2:** Nucleotide sequences of primers used in digital droplet PCR.

Target Name	Primer Sequence	References
miR398	F:GTATACTGTGTTCTCAGGTCG	This study
	R:GTGCAGGGTCCGAGGT	
miR167	F:CGCGATGAAGCTGCCAGCAT R:CAGTGCAGGGTCCGAGGT	[61]
miR159	F: CGCGCTTTGGATTGAAGGGA	[61]
	R:CAGTGCAGGGTCCGAGGT	
ADP-ribosyltransferase	F:GCTCTCCAACAACATTGCCAAC R: GCTTCTGCCTGTCACATACGC	[65]

### 4.11. Results Visualization

Graphical presentation of the results was performed using R version 3.6.3. [62] and the ggplot2 package [66].

## 5. Conclusions

Our research showed that miR398, miR167, and miR159 participate in the wheat–*F. culmorum* interaction and demonstrate time-, organ-, and fungal strain-specific expression patterns. The plant response at the miRNA level during fungal infection was higher in the early stage of *F. culmorum* infection and dynamically decreased a few days after inoculation, which may suggest a role of miR398, miR167, and miR159 in neutralizing both hormonal imbalances and oxidative stress caused by fungal invasion and, consequently, initiating a host defense response by means of the signaling pathway. Additionally, we observed that miR398, miR167, and miR159 were involved in the interactions of wheat with symbiotic *Trichoderma* and demonstrated their organ-, and species-specific expression patterns. Furthermore, we observed diverse expression patterns of studied miRNAs between *Trichoderma–*inoculated and *F. culmorum–*inoculated plants, as well as between *Trichoderma*–inoculated or *F. culmorum–*inoculated and control wheat plants. The preliminary research initiated here and the observations made confirm the need to continue work on the understanding of the role of miRNAs in both beneficial and deleterious interactions between wheat and fungi and determine their further directions. Thus, other important issues to focus on are correlating the observed abundance of miRNAs with the expression level of their target genes, and also examining the early stage of wheat–*Trichoderma* interaction. Further research is necessary due to the need to better understand the sophisticated miRNA regulation in the pathogenic *F. culmorum* invasion of wheat, as well as during the beneficial colonization of its tissues by *Trichoderma* fungi, which can ultimately be used to improve the condition, health, and productivity of this economically important crop.

## Figures and Tables

**Table 1:** Nucleotide sequences of studied miRNAs and primers used in stem loop pulsed RT-PCR.

miRNA (miRBase Accession No.)	miRNA Sequence	Stem Loop Primer Sequence
miR398 (MIMAT0018225)	uguguucucaggucgcccccg	GTCGTATCCAGTGCAGGGTCCGAGGTATTCGCACTGGATACGACCGGGGG
miR167 (MIMAT0005347)	ugaagcugccagcaugaucua	GTCGTATCCAGTGCAGGGTCCGAGGTATTCGCACTGGATACGACTAGATC
miR159 (MIMAT0005343)	uuuggauugaagggagcucug	GTCGTATCCAGTGCAGGGTCCGAGGTATTCGCACTGGATACGACCAGAGC

## Supplementary Materials

The following are available online at www.mdpi.com/article/10.3390/pathogens10111461/s1. Table S1. Results of expression comparison in early response of wheat’s miR398, miR167, and miR159 to *Fusarium culmorum* infection. Table S2. Results of expression comparison in late response of wheat’s miR398, miR167, and miR159 to *Fusarium culmorum* infection. Table S3. Results of comparison of the temporal response of wheat’s miR398, miR167, and miR159 level to *Fusarium culmorum* infection. Table S4. Results of comparison of Wheat’s miR398, miR167 and miR159 level to Inoculation of *Trichoderma* species and to the profiles formed by pathogenic *Fusarium* strains.
